# Pno1 Tissue-Specific Expression and Its Functions Related to the Immune Responses and Proteasome Activities

**DOI:** 10.1371/journal.pone.0046093

**Published:** 2012-09-28

**Authors:** Xuehai Wang, Tao Wu, Yan Hu, Martin Marcinkiewicz, Shijie Qi, Hector Valderrama-Carvajal, Hongyu Luo, Jiangping Wu

**Affiliations:** 1 Laboratoire d’immunologie, Centre de recherche, Centre hospitalier de l’Université de Montréal (CRCHUM)-Hôpital Notre-Dame, Montreal, Quebec, Canada; 2 Service de nephrologie, Centre de recherche, Centre hospitalier de l’Université de Montréal (CRCHUM)-Hôpital Notre-Dame, Montreal, Quebec, Canada; 3 Institute of Cardiology, First Affiliated Hospital, Zhejiang University Medical College, Hangzhou, China; 4 Cytochem Inc, Montreal, Quebec, Canada; University of Freiburg, Germany

## Abstract

Pno1 is a protein that plays a role in proteasome and ribosome neogenesis in yeast. So far, its functions in mammalian cells have not been investigated. To understand its function in mammals, we performed *in situ* hybridization analysis of Pno1 expression in different development stages and generated Pno1 gene knockout (KO) and transgenic (Tg) mice lineages. The results showed early lethality of homozygous Pno1 KO lineage caused, as demonstrated in parallel by ex vivo experiments, by arrest of embryo development before compaction stage. Though, heterozygous (HET) mice with 50% of normal Pno1 mRNA concentration were fertile and showed no obvious anomalies. The lymphoid organs of HET mice were normal in size, weight and cellularity, with normal T and B cell subpopulations. TCR-triggered activation and proliferation of HET T cells were normal. Proteasome activities in HET organs were uncompromised. Tg mice with actin promoter-driven Pno1 expression were also fertile, with no apparent anomalies, although they expressed 2–5-fold higher Pno1 mRNA levels. The lymphoid organs of Tg mice were of normal size, weight and cellularity with normal T and B cell sub-populations. TCR-triggered activation and proliferation of Tg T cells were normal. Tg organs and tissues presented normal proteasome activity as did their wild type counterparts. Tagged Pno1 over-expression in L cells and density gradient fractionation established that Pno1 existed in large complexes with sedimentation rates between 20S and 26S, bigger than mature 26S proteasomes. Pno1 in fractions did not coincide with 40S or 60S ribosome subunits. Our study indicates that Pno1 is essential for cellular functions, but only a small percentage of its normal level is sufficient, and excessive amounts are neither harmful nor useful. The nature of the large complexes it associates with remains to be identified, but it is certain that they are not mature proteasomes or ribosomes.

## Introduction

Ribosome neogenesis requires more than 200 assembly factors, most of which are not present in mature ribosomes. Many of these factors are needed at certain time points of ribosome maturation; they then dissociate from assembled intermediates and decay in an orderly fashion once their function is performed. Pno1 (partner of Nob1) is such a ribosome neogenesis factor. In yeasts, Pno1 is also called *Dim2, Rrp2* or *Yor145* [1]. It is highly conserved in yeasts up to mammalian species. Mouse *Pno1* mRNA comprises a 746-nt open-reading frame encoding a 248-amino acid peptide. Yeast and mouse Pno1 share 52% identity at their gene coding sequences and 46.7% homology (allowing amino acid substitution) at the protein level. Pno1 contains a K homolog (KH) domain that is capable of binding RNA [2]. Pno1 protein shows dynamic distribution during different phases of yeast growth from nucleolus to cytosol [3]. It is associated with Nob1 [4], which is involved in 90S to 40S pre-ribosome maturation [5] and is an exonuclease [1]. The KH domain of Pno1 is also essential for its binding to Nob1 [6]. Pno1 binds to both 90S and 40S pre-ribosomes [3] via Nob1. During pre-40S ribosome maturation, the kinases Rio2, Nob1 and Pno1 form complexes attached to the front of the late pre-40S ribosome head [7]. Pno1 increases Nob1 RNA affinity, and regulates Nob1’s cleavage activity at the 3′ end of 18S rRNA [6]. Loss of Pno1 results in accumulation of 35S, 33S and 32S rRNA [3] but with a decrease of 18S rRNA [8].

Pno1 and Nob1 are also critical for proteasome maturation in yeasts [4]. Again, and in this case, Pno1 interacts with Nob1 and both proteins form complexes with the 19S regulatory particle of 26S proteasomes. Mutation of either Pno1 or Nob1 causes defective assembly of 26S proteasomes [4].

**Figure 1:**
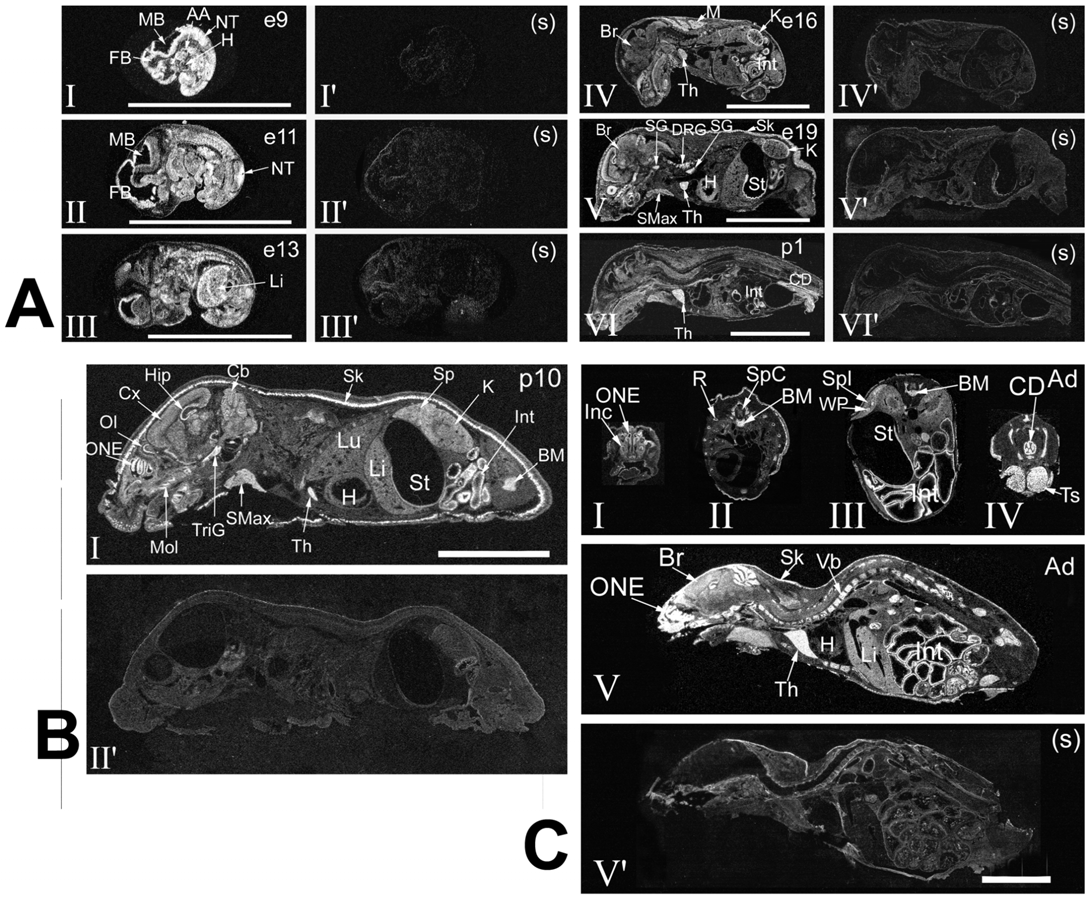
ISH analysis of Pno1 expression during ontogeny. AA: arch artery; Ad – adult; BM: bone marrow; Br: brain; Cb: cerebellum; CD: colon, descending; Cx: cerebral cortex; DRG: dorsal root ganglion; FB: forebrain; H: heart; Hip: hippocampus; Inc: incisors, teeth; Int: intestine; K: kidney; Li: liver; Lg: lacrimal gland; Lu: lungs; M: muscles, striated; MB: midbrain; Mol: molars, teeth; NT: neural tube; OL: olfactory lobe; ONE: olfactory neuroepithelium; R: rib; SG: sympathetic ganglion; Sk: skin; SMax: submaxillary gland; SpC: spinal cord; Spl: spleen; St: stomach; Th: thymus. TriG; trigeminal ganglion; Ts: testes; Vb: vertebrae. Bars = 1 cm. (s): sense cRNA used as a probe. The bars = 1 cm. Darkfield x-ray film autoradiography is shown. *A. Pno1 expression from embryonic day 9 (e9) to post-natal day 1 (p1)*. Panels I–VI: anti-sense cRNA as probe. Panels I’–VI’: sense cRNA as probe. *B. Pno1 expression on p10.* Panel I: anti-sense cRNA as probe. Panel I’: sense cRNA as probe. *C. Pno1 expression in adulthood*. Panels I–V: anti-sense cRNA as probe. Panel V’: sense cRNA as probe.

The above findings indicate that Pno1 performs important functions in both ribosome and proteasome biogenesis, but whether it undertakes both these functions at the same time or separately is not known.

**Figure 2:**
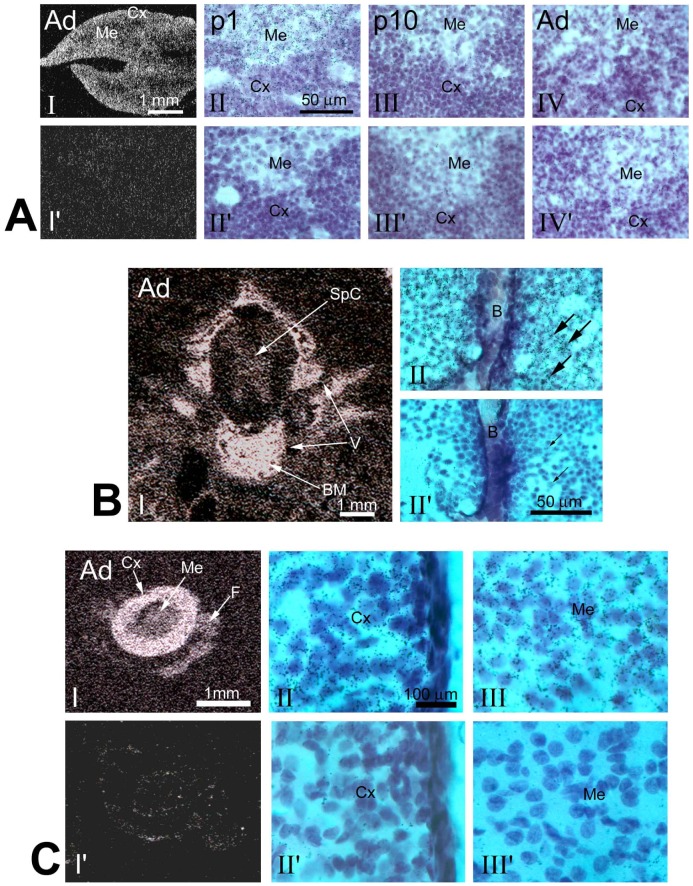
Pno1 expression in adult tissues according to ISH. Ad: adrenal gland; B: bone; Me: medulla; Cx: cortex; SpC: spinal cord; BM: bone marrow; Vb: vertebrae. (s): sense cRNA as probe. *A. Pno1 expression in Th* **.** Panel I: Dark field X-ray film autography with anti-sense cRNA as probe. Panel I’: sense cRNA as probe. Bar = 1 mm. Panels II to IV (antisense) and (II’ to IV’, sense) are brightfield images of emulsion autoradiography in p1, p10 and adult mice. *B. Pno1 expression in the vertebrae region of adult mouse whole-body section* **.** Panel I: Dark field X-ray film autography with anti-sense cRNA as probe. Panels I (anti-sense cRNA as probe) and II (sense cRNA as probe): emulsion autoradiography seen under brightfield illumination at higher magnification. Fine black grains represent Pno1 mRNA labelling. Bar = 1 mm (in I) and 50 µm (in II). Arrows point to small hematopoietic cells. *C. Pno1 expression in the Ad*. Panels I–III: Anti-sense cRNA as probe. Panels I’–III’: sense cRNA as probe. Panels I and I’ are dark field X-ray film autography. Panels II, III, II’ and III’ are emulsion autoradiography seen under higher magnification. Bar = 1 mm (in I) and 100 µm (in II).

To date, to the best of our knowledge, all functional and binding studies of Pno1 have been conducted in yeasts. The Pno1 expression pattern in different tissues and organs of mammals is unclear. It is uncertain whether Pno1 is vital and irreplaceable in mammalian cells or if redundancy exists so that a lack of Pno1 will not be fatal to cells and/or animals. It is also not known whether different Pno1 expression levels impede cellular functions. In this study, we employed *in situ* hybridization (ISH), gene knockout (KO) and transgenic (Tg) over-expression in mice to address these questions.

**Figure 3:**
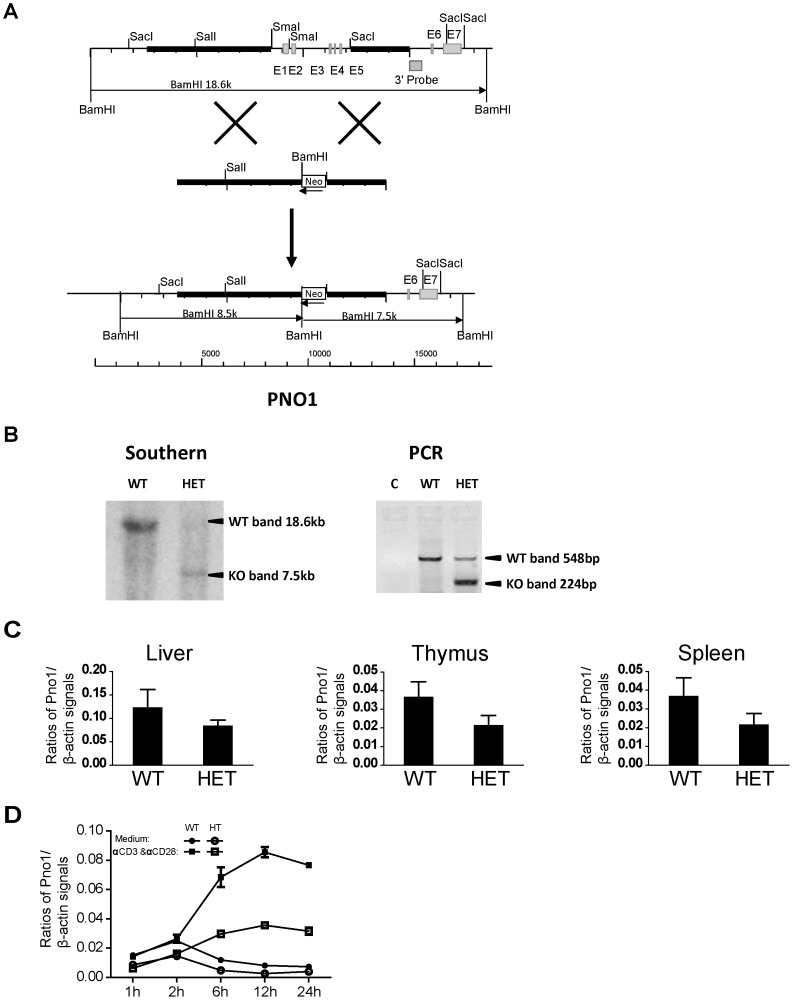
Generation of Pno1 KO mice. *A. Targeting strategy to generate Pno1 KO mice.* The black square marked as 3′ probe represents the sequence used as probe in Southern blotting for genotyping. A 18.6-kb BamH1 fragment detected by this probe represents the WT allele, and a 7.5-kb BamH1 fragment, the KO allele. *B. Genotyping of Pno1 mutant mice* **.** Tail DNA was digested with BamHI, and analyzed by Southern blotting (left panel), with a 3′ probe whose sequence location is indicated in A. A 18.6-kb band representing the WT allele and a 7.5-kb band representing the recombinant allele are indicated by arrows. Ear lobe DNA without digestion was analyzed by PCR for routine genotyping (right panel). A 548-bp band representing the WT allele and a 224-bp band representing the recombinant allele are indicated by arrows. *C. Reduced Pno1 mRNA expression in Pno1^+/−^ tissues*. mRNA from the Li, Th and Spl of WT and heterozygous Pno1^+/−^ (HET) mice were analyzed by reverse transcription-real time PCR (RT-qPCR) for Pno1 mRNA levels. The results are expressed as ratios of Pno1 versus β-actin signals with means ± SD indicated. *D. Reduced Pno1 mRNA up-regulation in Pno1^+/−^ T cells upon activation*. T cells from WT and HET Spl were stimulated with solid phase anti-CD3 mAb and anti-CD28 mAb (0.5 µg/ml and 4 µg/ml respectively for coating) for 1 to 24 h, and their Pno1 mRNA levels were quantified by RT-qPCR. The results are expressed as ratios of Pno1 versus β-actin signals with means ± SD indicated.

## Materials and Methods

### In situ Hybridization

Full-length 1526-bp Pno1 cDNA in pSPORT1 (clone H3085H06; accession number NM_025443) from National Institute of Aging, USA) was employed as a template for sense and anti-sense riboprobe synthesis, using SP6 and T7 RNA polymerase for both ^35^S-UTP and ^35^S-CTP incorporation [9].

Tissues from WT mice were frozen in −35°C isopentane, and kept at −80°C until sectioned. ISH, x-ray film and emulsion autoradiography was performed using 10-µm thick cryostat-cut slices, as outlined previously [9].

**Figure 4:**
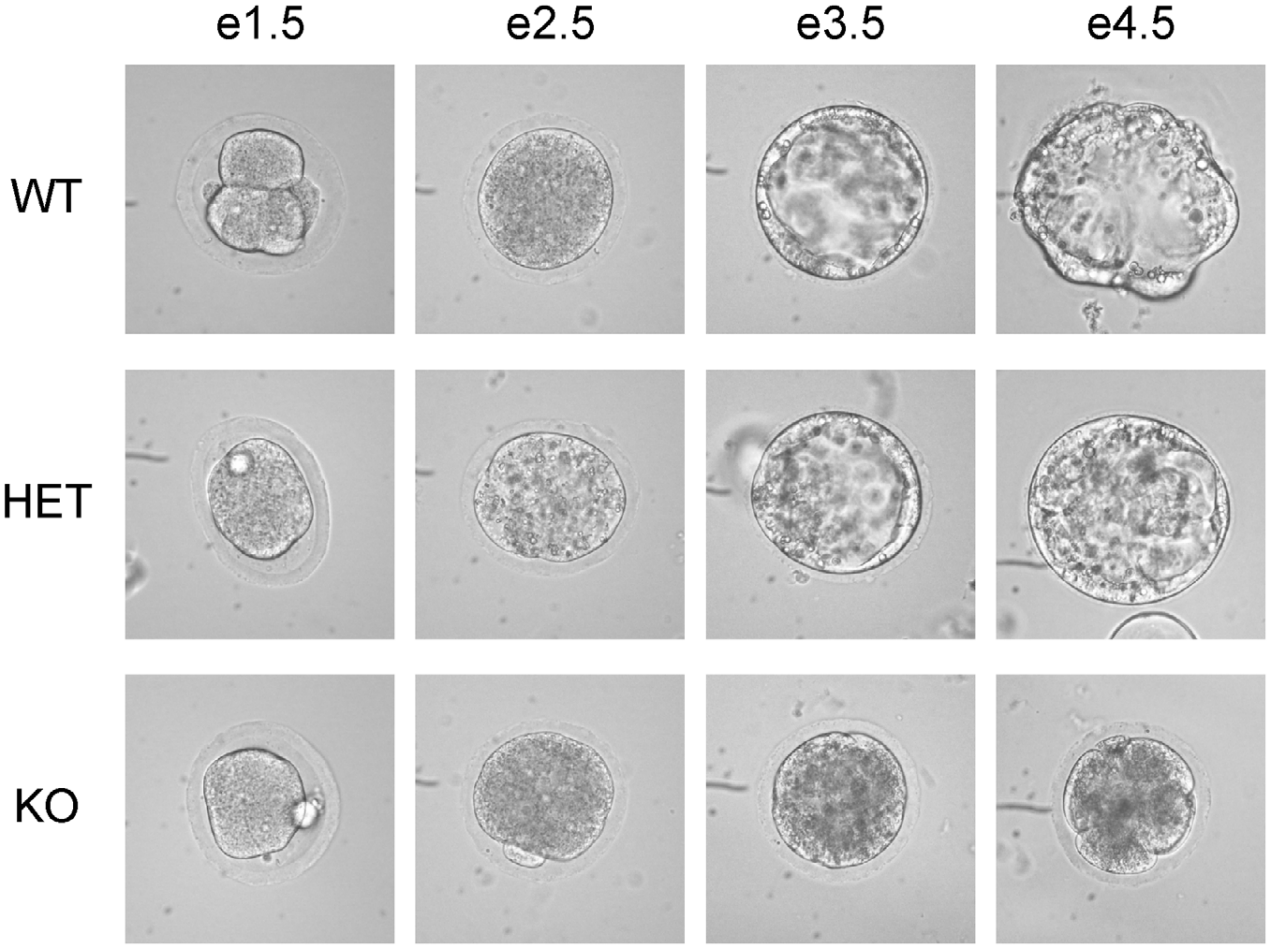
In vitro development of WT, HET and KO embryos. e1.5 embryos were harvested, and cultured in M16 medium and photographed daily until e4.5. Phase contrast micrographs are shown. At the end of culture on e4.5, the embryos were genotyped with qPCR.

**Table 1:** Genotypic analysis of embryos from PNO1^+/−^ × PNO1^+/−^ mating.

	Genotype			
Stage	+/+	+/−	−/−	Total
e6.5	6	12	0	18
e3.5	8	15	4	27

### Generation of Pno1 Gene Knockout (KO) Mice

A polymerase chain reaction (PCR) fragment, amplified on the Pno1 cDNA sequence (clone H3085H06), was used as probe to isolate genomic BAC DNA clone 115L21 from the 129/sv mouse BAC genomic library RPCI-22. Targeting vectors were constructed by recombination and routine cloning methods with a 12.5-kb Pno1 genomic fragment from clone. A 3.6-kb SmaI-SaclI genomic fragment containing exons 1 to 5 was replaced by a 1.1-kb Neo cassette from pMC1Neo Poly A. The final targeting fragment was excised from its cloning vector backbone by Not I digestion and electroporated into R1 embryonic stem (ES) cells for G418 selection [10]. The targeted ES cell clones were injected into C57BL/6 blastocysts. Chimeric male mice were mated with C57BL/6 females to establish mutated Pno1 allele germline transmission. All mice were housed under specific pathogen-free conditions and used in accordance with a protocol approved by the Institutional Animal Protection Committee of the University of Montreal Hospital Center (NO9055JWs).

**Figure 5:**
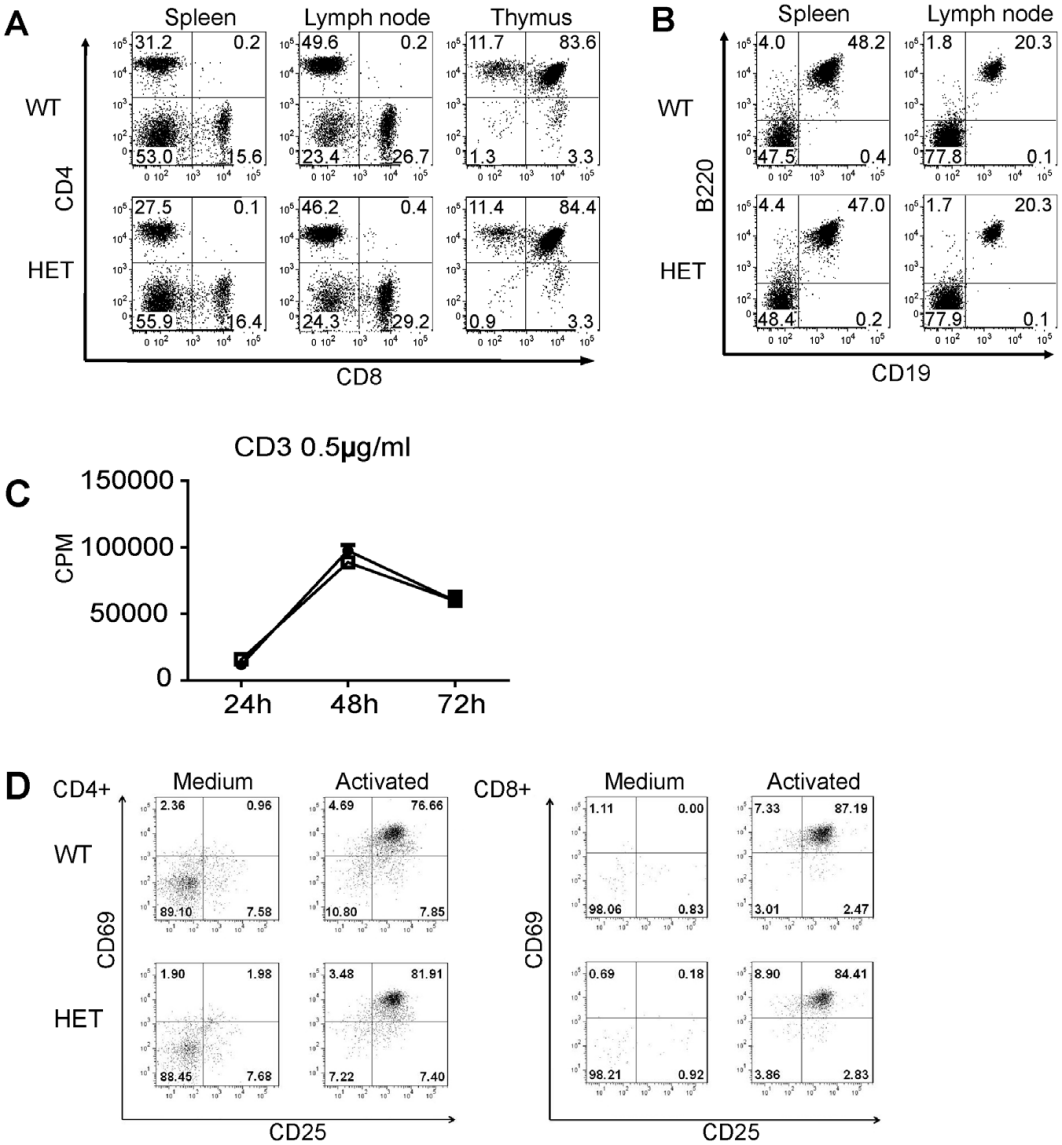
Sub-populations of lymphocytes in lymphoid organs and T cell function of Pno1 HET mice. A. T cell subpopulations in the spleen and LN. CD4 and CD8 T-cell populations in WT and HET spleens, LN and thymuses were analyzed by 2-color flow cytometry. Percentages are indicated. B. B cell population in lymphoid organs. B cell populations in the spleen and LN were analyzed according to B220 and CD19 expression by 2-color flow cytometry. C. T cell proliferation. WT and HET spleen T cells were stimulated with solid phase anti-CD3 mAb (0.5 µg/ml for coating). The cells were pulsed with ^3^H-thymidine 16 h before harvesting. ^3^H-thymidine uptake by the cells was measured at 24, 48 and 72 h. Samples were tested in triplicate, and means ± SD of CPM are shown. D. C69 and CD25 expression on activated WT and HET T cells. WT and HET T cells were stimulated overnight by solid phase anti-CD3 mAb plus anti-CD28 mAb (0.5 µg/ml and 4 µg/ml respectively for coating). CD69 and CD25 expression on CD4 (left panel) and CD8 (right panel) T cells was measured by 2-color flow cytometry. All experiments in this figure were repeated at least 3 times and representative data are reported.

Southern blotting with a probe corresponding to 3′ sequences outside the targeting region screened for and confirmed gene targeting in ES cells and eventually in mouse tail DNA. The targeted allele showed a 8.5-kb BamHI/BamHI fragment, while the wild type (WT) allele had an 18.6-kb BamH1/BamHI fragment. PCR was adopted for routine genotyping of the targeted allele(s). The following PCR conditions were applied: 4 min at 95°C, followed by 35 cycles of 30 s at 94°C, 30 s at 58°C, and 60 s at 72°C, with final incubation at 72°C for 10 min. Two forward primers (5′- CTGCGTGTTCGAATTCGCCAATGA-3′, forward primer 1; 5′-ACCACCTGTCAAG GGCAATAGGAA-3′, forward primer 2) and 1 reverse primer (5′- TTCACCTAGAACCAC ATG CCCACA-3′) were included in the PCR, which amplified a 224-bp fragment from the targeted allele and a 548-bp fragment from the WT allele.

**Figure 6:**
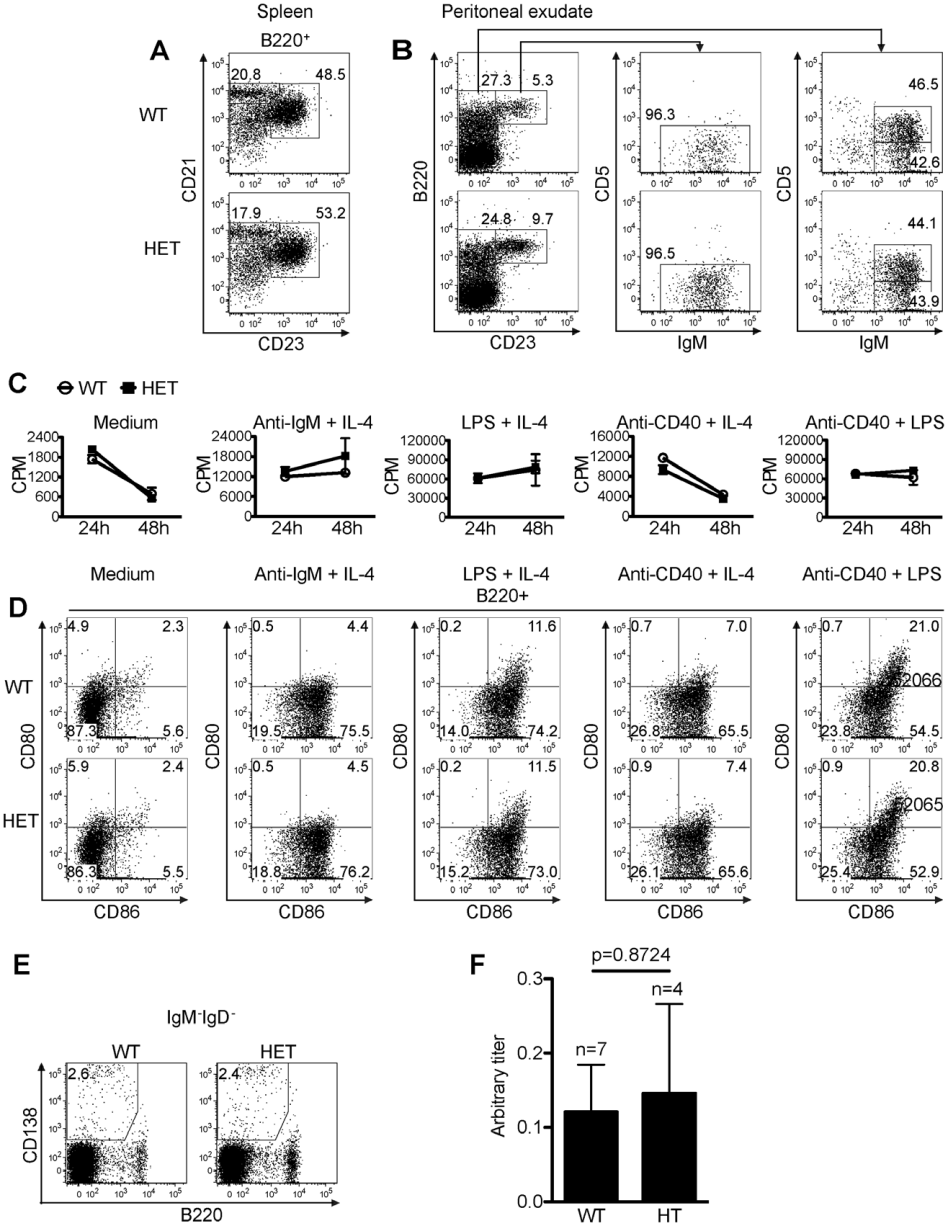
B cell development and function in HET mice. *A. Normal marginal zone B cell and follicular B cell populations in the spleen of HET mice.* Marginal Zone B cell (B220^+^CD21^hi^CD23^low/−^) and follicular B cell (B220^+^CD21^int/hi^CD23^hi^) populations in WT and HET spleens were analyzed by 3-color flow cytometry. Percentages are indicated. *B. B1 B cell and B2 B cell subpopulation in peritoneal exudates of HET mice.* Peritoneal exudate B1a (B220^+^CD23^−^CD5^+^IgM^hi^), B1b (B220^+^CD23^−^CD5^−^IgM^hi^) and B2 (B220^+^CD23^+^IgM^int/hi^) B cells of WT and HET mice were analyzed by by 4-color flow cytometery. Percentages are indicated. *C. Proliferation of B cells from HET mice.* WT and HET spleen B cells were stimulated with different stimuli as indicated (anti-IgM: 5 µg/ml; IL-4∶10 ng/ml; anti-CD40 mAb: 2 µg/ml; LPS: 2 µg/ml). The cells were pulsed with ^3^H-thymidine 6 h before harvesting. ^3^H-thymidine uptake by the cells was measured at 24 h and 48h after the initiation of the culture. Samples were in triplicate, and means ± SD of CPM are shown. *D. B cell activation markers CD80 and CD86 expression on activated WT and HET B cells*. WT and HET spleen B cells were stimulated as described in *C*. CD80 and CD86 expression on B220^+^ B cells was measured by 3-color flow cytometry 24 h after the initiation of the culture. *E. Plasma cells in the draining lymph nodes of immunized WT and HET mice*. WT and HET mice were immunized with chick type II collagen with adjuvants at the tail base and sacrificed 21 days after the immunization. Isotype-switched plasmablast/plasma cells (IgD^−^IgM^−^CD138^+^B220^lo/−^) from the draining lymph nodes of WT and HET were analyzed by 4-color flow cytometry. *F. Serum collagen-specific antibody production in WT and HET mice*. Sera from mice (WT n = 7, HET n = 4) as described in E were collected on day 21 after the immunization. Chick collagen-specific IgG Abs were measured by ELISA. The data are expressed as arbitrary titres. The titres between WT and HET groups were not statiscially significant (p = 0.8724, Student’s t test). Experiments A-E were repeated at least 3 times and representative data are shown.

### Generation of Pno1 Tg Mice

1,526-bp mouse full-length Pno1 cDNA was excised from pSPORT1 with Not I/Sal I, blunt-ended, and cloned into blunt-ended BamH I/Xba I sites in vector pAC, between the human β-actin promoter and β-actin polyA signals. The resulting construct was named pAC-Pno1. The 6.4-kb ClaI/ClaI fragment containing the β-actin promoter, Pno1 cDNA and β-actin polyA signals was excised and injected into fertilized C3H×C57BL/6 eggs. Tg mice were genotyped by PCR with tail DNA under the following conditions: 4 min at 95°C, followed by 30 cycles of 30 s at 94°C, 30 s at 58°C, and 30 s at 72°C, with final incubation at 72°C for 10 min. Primers 5′-GTCATGGCAGAAACTTGCAC-3′ (forward) and 5′- GAATGCAATTGTTGTTGGTAACTTG-3′ (reverse) amplified a 552-bp product.

**Figure 7:**
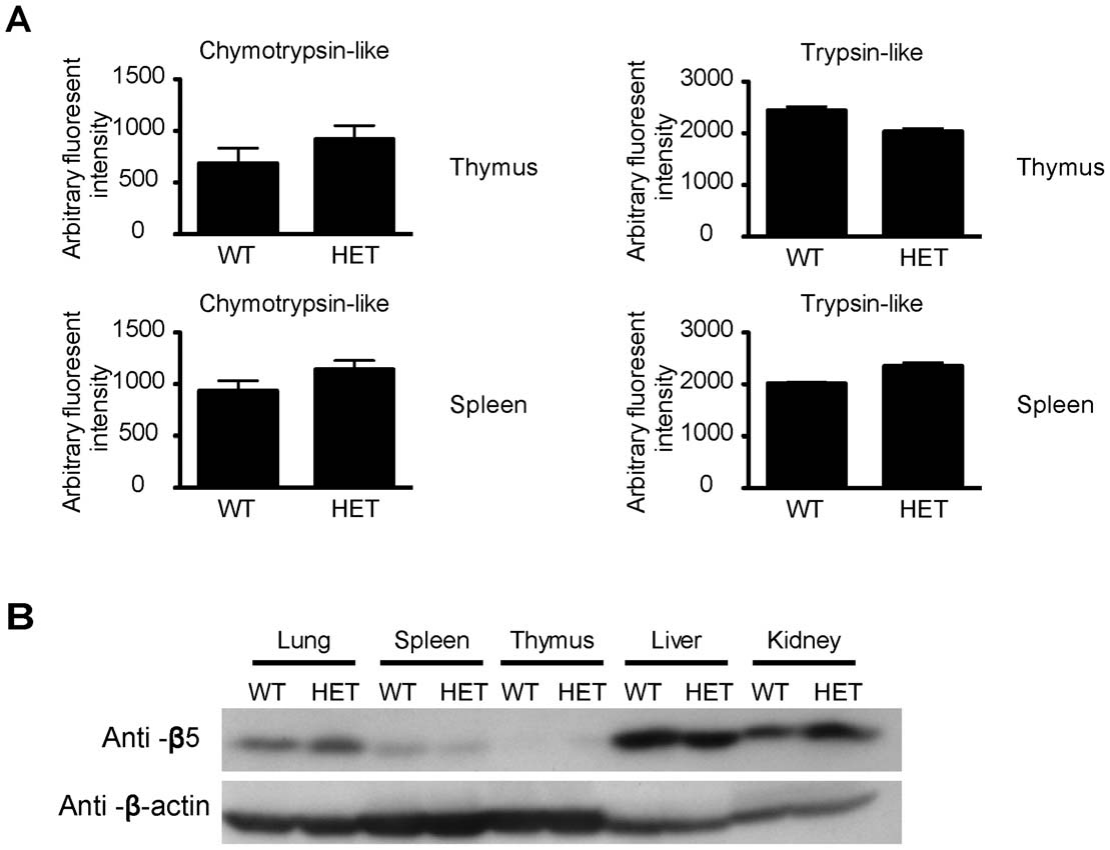
Proteasome activities WT and HET tissues. *A. Proteasome activities in spleen and thymus.* Lysates from the Sp and Th of WT and HET mice were assayed for chymotrypsin-like and trypsin-like proteasome protease activities. Samples were tested in triplicate, and means ± SD are shown. *B. HET and WT organs have similar proteasome levels.* Lysate proteins from the lung, spleen, thymus, liver and kidney were analyzed for proteasome content according to proteasome β5 subunit levels based on immunoblotting (upper panel). β-actin levels were used to show even loading of lysate proteins (lower panel).

### Reverse Transcription and Real-time PCR (RT-qPCR)

Pno1 mRNA in cells or tissues from KO, Tg and WT mice was measured by RT-qPCR. Total RNA from cells was extracted with TRIzol® (Invitrogen, Carlsbad, CA, USA) and then reverse-transcribed with Superscript II™ reverse-transcriptase (Invitrogen). The forward and reverse primers were 5′-TGTTCTTGGCTTTCAGGTGGAGGA-3′ and 5′-TTCCTATTGCCC TTGACAGGTGGT-3′, respectively. A 125-bp product was detected with the following amplification program: 95°C×15 min, 1 cycle; 94°C×15 s, 55°C×30 s, 72°C×30 s, 40 cycles.

β-actin mRNA levels were measured as internal controls; the forward and reverse primers were 5′-TGGTACCACAGGCATTGTGAT-3′ and 5′-TGATGTCACGCACGATTTCCCT-3′, respectively, with the same amplification program as for Pno1 mRNA.

qPCR was performed in triplicate, and the results were expressed as the signal ratios of Pno1/β-actin.

**Figure 8:**
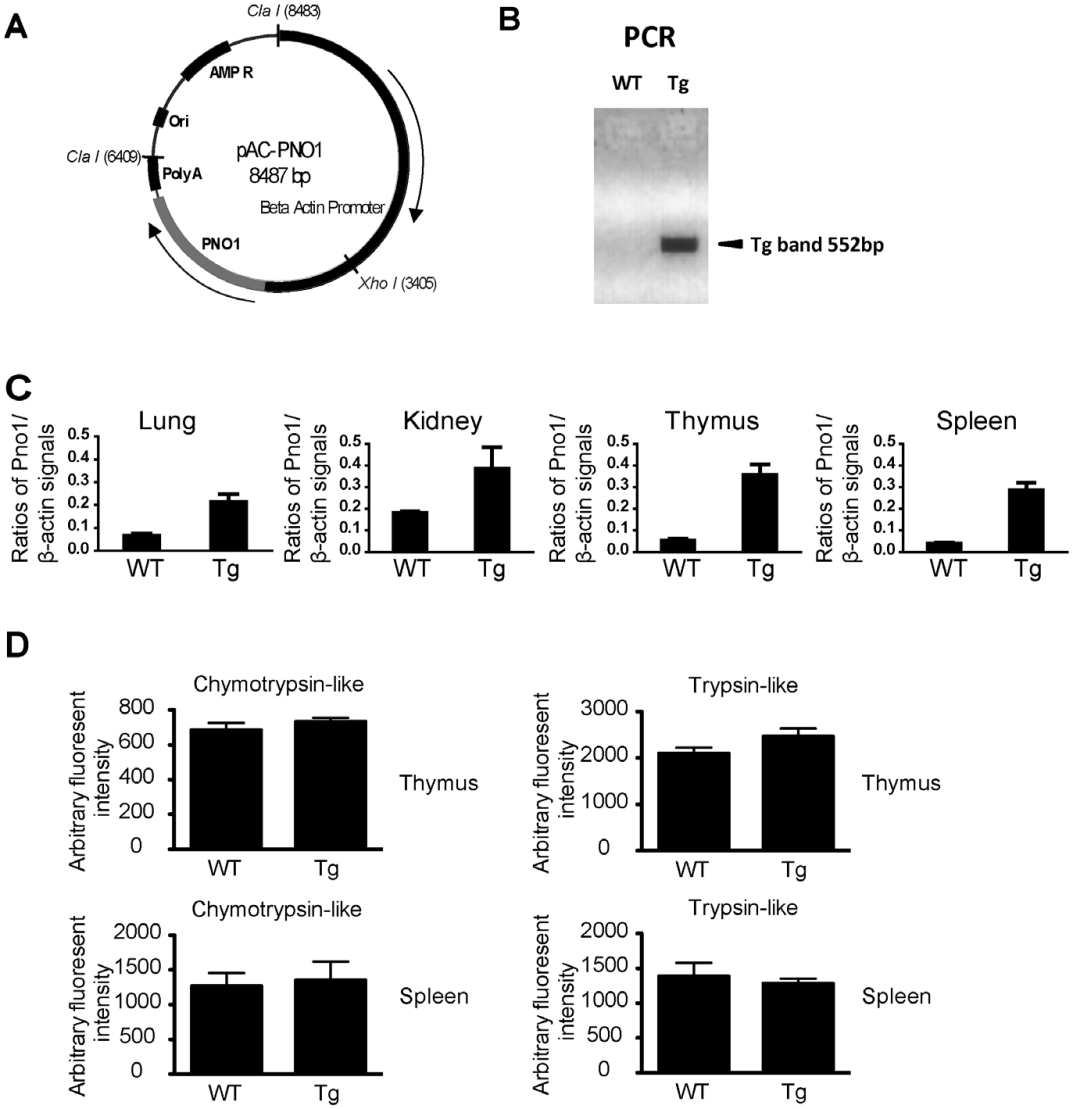
Generation of Pno1 Tg mice. *A. pAC-Pno1 construct for Tg mice generation* **.** The 6.4-kb ClaI/ClaI fragment was microinjected. *B. Genotyping of ear lobe DNA by PCR*. The 552-bp band specific to the Pno1 transgene is indicated by an arrow. *C. RT-qPCR of Pno1 mRNA in different organs of Tg mice*. Means ± SD of Pno1 versus β-actin signal ratios of the lung, kidney and spleen of Tg and WT mice. *D. Proteasome activities in spleen and thymus* **.** Lysates from the Sp and Th of WT and Tg mice were assayed for chymotrypsin-like and trypsin-like proteasome protease activities. Samples were tested in triplicate, and means ± SD are shown.

qPCR was also employed for embryo genotyping. Embryos were digested at 55°C for 4 h in 2 µl digestion buffer (proteinase K, 0.01% gelatin, 0.005% NP-40, 20 mM Tris (pH 8.35), 40 mM KCl, 0.5 mM MgCl_2_). Forward primer 5′-GGTTTGCTCGACATTGGGGTGGAAA-3′ and reverse primer 5′-AGCGCGAGTATTCACCTAGAACCA-3′ detected a 185-bp fragment from the KO allele. Forward primer 5′-GGTCCAAGAACGTTGCCAGGAAAT-3′ and reverse primer 5′-AGGGTCTGATTCCTCAATGCTCCA-3′ detected a 168-bp fragment from the WT allele. PCR conditions for the reactions were as follows: 50°C×2 min, 1 cycle; 95°C×2 min, 1 cycle; 94°C×10 s, 58°C×20 s, 72°C×20, 35 cycles.

**Figure 9:**
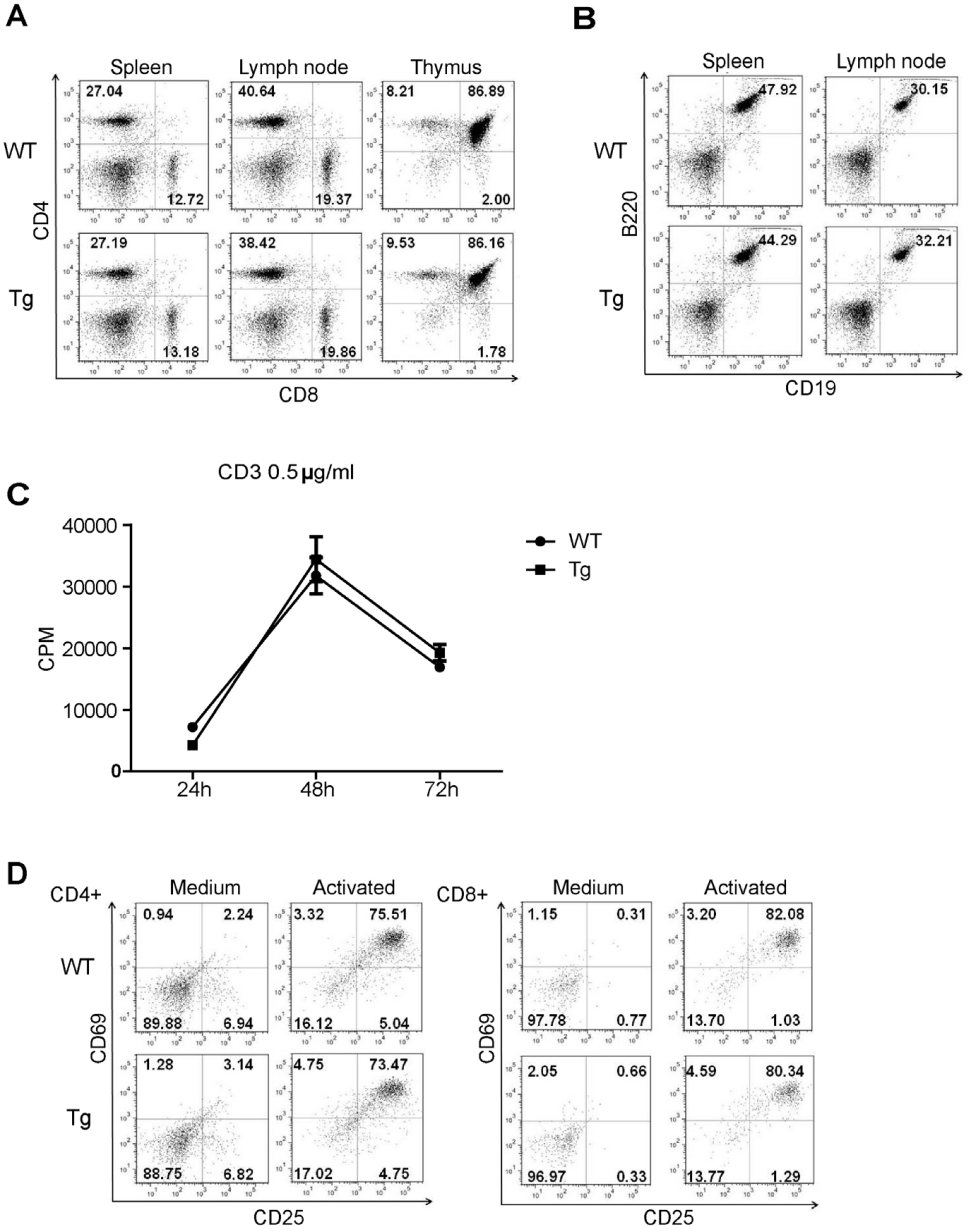
Sub-populations of lymphocytes in lymphoid organs and T cell function of Pno1 Tg mice. A. T cell sub-populations in the spleen and LN. CD4 and CD8 T cell populations in the WT and Tg spleen LN and thymus were analyzed by 2-color flow cytometry. Percentages are indicated. B. B cell population in lymphoid organs. The B cell population in the spleen and LN was analyzed according to B220 and CD19 expression by 2-color flow cytometry. C. T cell proliferation. WT and Tg spleen T cells were stimulated with solid phase anti-CD3 mAb (0.5 µg/ml for coating). The cells were pulsed with ^3^H-thymidine 16 h before harvesting. ^3^H-thymidine uptake by cells was measured at 24, 48 and 72 h. The samples were tested in triplicate, and means ± SD of CPM are shown. D. C69 and CD25 expression on activated WT and HET T cells.WT and Tg T cells were stimulated overnight by solid phase anti-CD3 plus anti-CD28 mAbs (0.5 µg/ml and 4 µg/ml respectively for coating). CD69 and CD25 expression on CD4 (left panel) and CD8 (right panel) T cells was measured by 2-color flow cytometry. All experiments in this figure were repeated at least 3 times and representative data are shown.

### Flow Cytometry

Single cell suspensions from the thymus, spleen and in certain experiments the draining lymph nodes were prepared and stained for flow cytometry, as described in our previous publications [11], [12].

### T and B Cell Proliferation

T cell proliferation was assessed by ^3^H-thymidine uptake, as detailed elsewhere [13].

### Mouse Immunization and ELISA

Eight- to 10-wk old WT, HET and Tg mice were immunized at the base of the tail with 100 µg of chick type II collagen (Chondrex, Redmond, WA), which was emulsified in equal volumes of Freund’s complete adjuvant (4 mg/ml Mycobacterium tuberculosis, strain H37Ra; Difco, Detroit, MI). On day 21, the mice were sacrificed and sera were collected for collagen-specific Ab assays [14].

**Figure 10:**
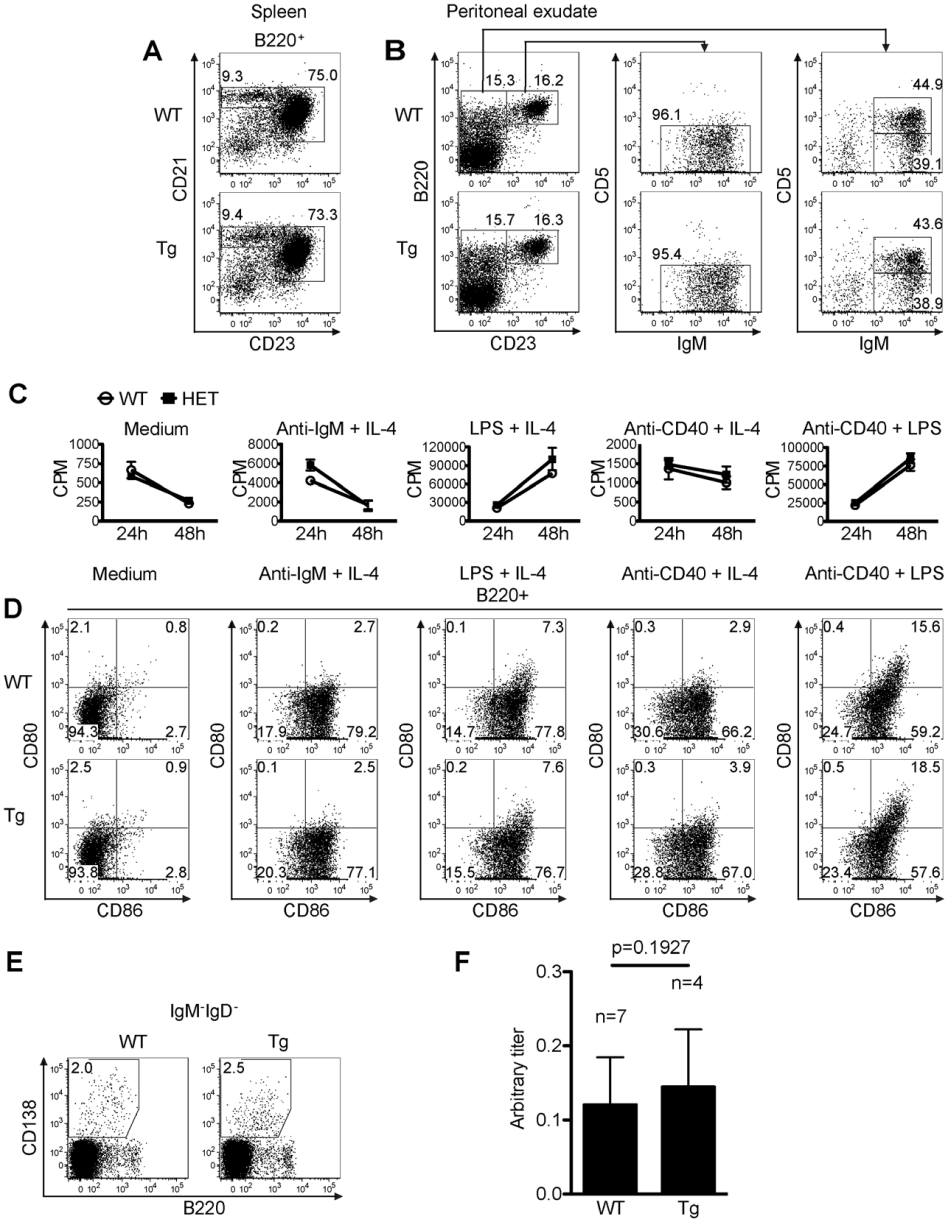
B cell development and function in HET mice. *A. Normal marginal zone B cell and follicular B cell populations in the spleen of Tg mice.* Marginal Zone B cell (B220^+^CD21^hi^CD23^low/−^) and follicular B cell (B220^+^CD21^int/hi^CD23^hi^) populations in WT and Tg spleens were analyzed by 3-color flow cytometry. Percentages are indicated. *B. B1 B cell and B2 B cell subpopulation in peritoneal exudates of Tg mice.* Peritoneal exudate B1a (B220^+^CD23^−^CD5^+^IgM^hi^), B1b (B220^+^CD23^−^CD5^−^IgM^hi^) and B2 (B220^+^CD23^+^IgM^int/hi^) B cells of WT and Tg mice were analyzed by by 4-color flow cytometery. Percentages are indicated. *C. Proliferation of B cells from Tg mice*. WT and Tg spleen B cells were stimulated with different stimuli as indicated (anti-IgM: 5 µg/ml; IL-4∶10 ng/ml; anti-CD40 mAb: 2 µg/ml; LPS: 2 µg/ml). The cells were pulsed with ^3^H-thymidine 6 h before harvesting. ^3^H-thymidine uptake by the cells was measured at 24 h and 48 h after the initiation of the culture. Samples were in triplicate, and means ± SD of CPM are shown. *D. B cell activation markers CD80 and CD86 expression on activated WT and Tg B cells*. WT and Tg spleen B cells were stimulated as described in *C*. CD80 and CD86 expression on B220^+^ B cells was measured by 3-color flow cytometry 24 h after the initiation of the culture. *E. Plasma cells in the draining lymph nodes of immunized WT and Tg mice*. WT and Tg mice were immunized with chick type II collagen with adjuvants at the tail base and sacrificed 21 days after the immunization. Isotype-switched plasmablast/plasma cells (IgD^−^IgM^−^CD138^+^B220^lo/−^) from the draining lymph nodes of WT and Tg were analyzed by 4-color flow cytometry. *F. Serum collagen-specific antibody production in WT and Tg mice* **.** Sera from mice (WT n = 7, Tg n = 4) as described in E were collected on day 21 after the immunization. Chick collagen-specific IgG Abs were measured by ELISA. The data are expressed as arbitrary titres. The titres between WT and Tg groups were not statiscially significant (p = 0.1927, Student’s t test). Experiments A-E were repeated at least 3 times and representative data are shown.

### Proteasome Activities

Lysates from WT, Tg, and heterozygous (HET) mouse thymus and spleen cells were assayed for 3 major protease activities (chymotrypsin-like activity, trypsin-like activity, and caspase-like activity) of proteasomes based on enzymatic digestion of fluorogenic substrates (Suc-LLVY-AMC for chemotrypsin-like activity, Z-LLA-AMC for trypsin-like activity, and Z-LLG- βNA for caspase-like activity). 20 µg of lysate protein were reacted with the substrates (100 nM) in 100 µl buffer containing 100 mM Tris-HCl (pH 8.2) at 37°C for 20 min. The reaction was stopped by adding 4 µl 2M HCl, and fluorescence intensity of the reaction solution was measured in a BioTek Synergy™ 4 Hybrid Microplate Reader with excitation at 380 nm and emission at 460 nm for AMC-conjugated substrates, and with excitation at 335 nm and emission at 410 nm for Z-LLG- βNA.

### Pno1 Overexpression in L Cells

1,526-bp mouse full-length Pno1 cDNA was inserted downstream of the cytomegalovirus (CMV) promoter and hemagglutinin (HA) tags in mammalian expression vector pCEP4-HA. The construct, named pCEP4-Pno1-HA, drove HA-tagged Pno1 over-expression in mammalian cells. L cells were transfected by pCEP4-Pno1-HA with Lipofectamine followed by Hygromycin B selection.

### Protein Fractionation by Glycerol Density Gradients

About 60 million pCEP4-Pno1-HA-transfected L cells were sonicated in 1 ml lysis buffer (25 mM Tris, pH 7.6, 10 µg/ml PMSF, 1 µg/ml LA, 1 mM DTT, 2 mM ATP, and 2 mM MgCl_2_). The lysates were centrifuged at 12,000*g* for 30 min at 4°C to remove debris. The cleared supernatants (700 µl) were laid on top of a 15-ml 10–40% glycerol density gradient (diluted in lysis buffer) in a 15-ml centrifuge tube and centrifuged at 83,000*g* for 22 h at 4°C in a Beckman SW28 rotor. The gradient was separated into thirty 0.5-ml fractions for further tests.

### Immunoblotting

200-µl aliquots from each gradient fraction were mixed with 800 µl cold acetone and stored at −20°C for 60 min to precipitate proteins dissolved in 30 µl RIPA buffer (25 mM Tris, pH 7.6, 150 mM NaCl, 1% NP-40, 1% sodium deoxycholate, 0.1% SDS) supplemented with protease inhibitors (Complete™ Protease Inhibitor Cocktail, Roche Diagnostics, Laval, Quebec, Canada) and centrifuged for 20 min at 12,000*g* at room temperature. Total supernatants were resolved by 12% SDS-PAGE and transferred to nitrocellulose membranes. The proteasome β5 subunit and β-actin were detected by blotting with rabbit anti-human β5 antibody (Ab, Millipore, Billerica, MA, USA), followed by horse radish peroxidase-conjugated goat anti-rabbit IgG (GE Healthcare, Little Chalfont, Bucks, United Kindom.). The membranes were then stripped and re-probed with horse radish peroxidase-conjugated rabbit anti- β-actin Ab (#4967, Cell Signaling Technology, Danvers, MA). Signals were detected with SuperSignal West Pico Chemiluminescent Substrate (Thermo Scientific, Rockford, IL, USA).

**Figure 11:**
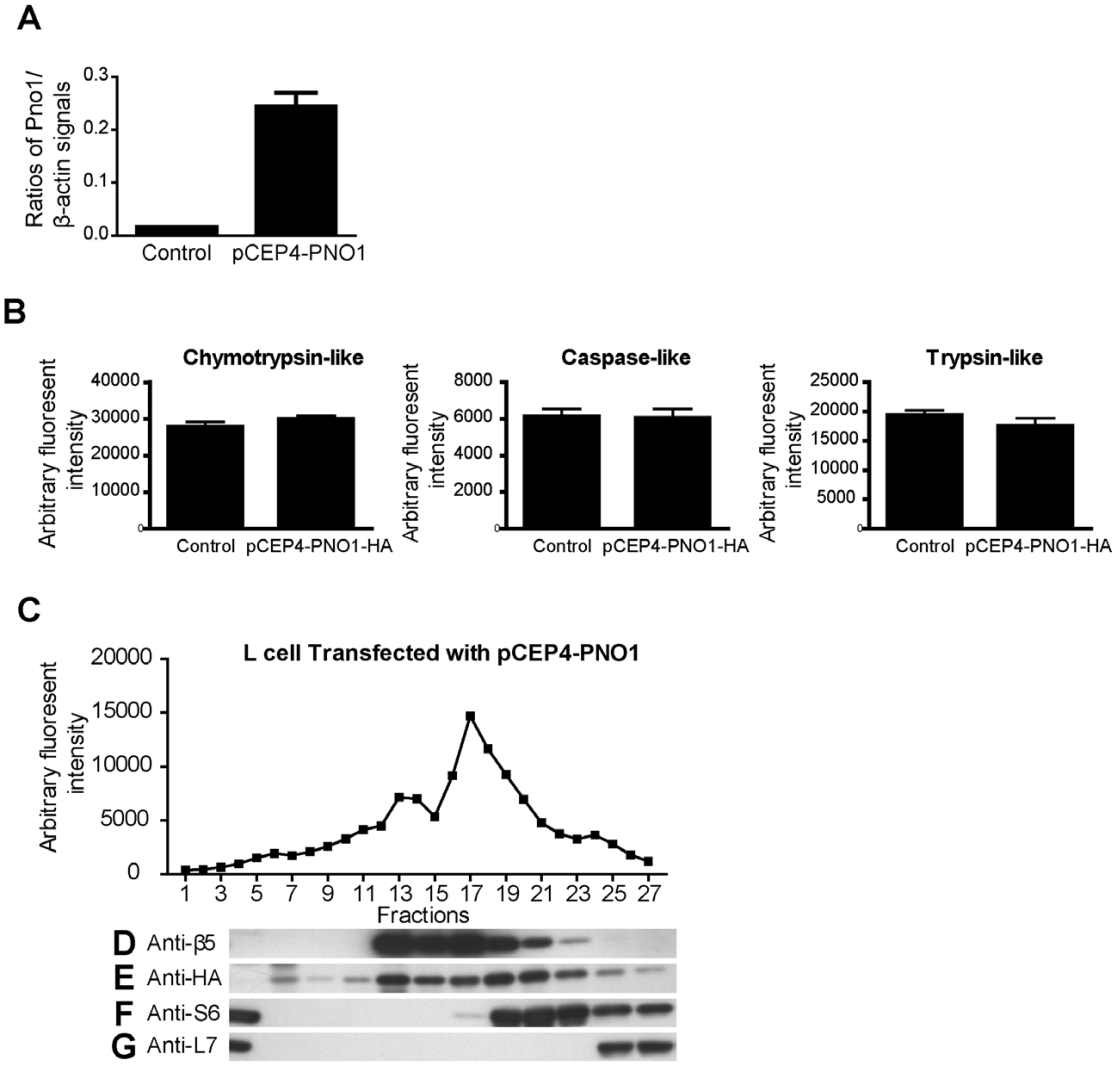
Pno1 is associated with macromolecules with a sedimentation rate greater than 26S. *A. Pno1 mRNA over-expression in L cells stably transfected with the Pno1-expressing construct pCEP-Pno1-HA* **.** Pno1 mRNA levels were quantified by RT-qPCR. Samples were tested in triplicate and means ± SD of Pno1 versus β-actin signal ratios are shown. L cells stably transfected with empty vectors were used as controls. *B. L cells over-expressing Pno1 presented similar proteasome activity as vector-transfected cells* **.** The chymotrypsin-like activity of L cells stably transfected with pCEP-Pno1-HA was quantified, and L cells transfected with empty vectors were used as controls. The assay was conducted in triplicate, and means ± SD are shown. *C. Proteasome activities in glycerol density gradient fractions* **.** Lysates from L cells stably transfected with Pno1-expressing constructs or empty vectors were fractionated with glycerol gradients, and chymotrypsin-like proteasome activity in fractions 1 to 27 was quantified. *D-G. Location of Pno1, β5, S6 and L7 in glycerol density gradients* **.** Proteins in fractions 1 to 27 of the glycerol gradient of L cells overexpressing Pno1 were analyzed by immunoblotting. The β5 proteasome subunit (D), Pno1 (E), S6 subunit of the small ribosome complex (F) and L7 subunit of the large ribosome complex (G) were detected by their respective Abs (Pno1 was detected by anti-HA Ab).

## Results

### Pno1 Expression Pattern in WT Mice According to in situ Hybridization

Based on the exposure time (optimal time: 4 days) necessary to produce X-Ray film autoradiogram, Pno1 mRNA belonged to a class of low abundant mouse mRNAs.

At mid-gestation embryo on day 9 (e9), Pno1 signals in most tissues, including the rudimental brain (MB: midbrain; FB: forebrain; NT: neural tube) and cardiovascular system (H: heart; AA: aortic arch), were relatively high, as illustrated in Figure 1A–I. After this stage and at subsequent gestation ages, density and spatial hybridization patterns underwent substantial change. The large cerebral Pno1-positive area was reduced to the periventricular band region on e11 and e13 (Figs. 1A–II and 1A–III). Pno1 mRNA level in the liver (Li) was moderate to high on e13 (Fig. 1A–III), but dropped significantly on e16 (Fig. 1A–IV). In contrast, Pno1 mRNA signals became perceptible in the kidneys (K) and Th, starting on e16 (Fig. 1A–IVD). Peak of Pno1 expression was observed in developing striated muscles (M) and intermingled adipose tissue on e16 (Fig. 1A–IV). Heart ventricles on e19 transiently displayed Pno1 mRNA levels (Fig. 1A–V) higher than on e9 (panel H in Fig. 1A–I). Around parturition (e19 and post-natal day 1 [p1]), Pno1 mRNA was clearly present in the immune system such as the thymus (Th), the digestive system such as the submaxillary gland (SMax), stomach (St), intestine (Int) and colon (descending) (CD)), peripheral nervous system (dorsal root ganglia (DRG), sympathetic ganglia (SG)), and skin (Sk) (Figs. 1A–V and 1A–IV). However, Pno1 mRNA signals were only slightly above background in the remaining tissues.

The Pno1 expression pattern on p10 remained similar to that on p1 (Fig. 1A–VI). Several particular brain regions, such as the olfactory lobe (OL), olfactory neuroepithelium (ONE) and hippocampus (Hip), manifested high expression levels; signals in the molars (Mol), trigeminal ganglion (TriG), Int and bone marrow (BM) were also elevated; the spleen displayed moderate expression (Fig. 1B).

In adulthood, Pno1 expression was noted in teeth, such as the incisors (Inc) (Fig. 1C–1), vertebrate bone marrow (Vb) (Figs. 1C–II and V) and testes (Ts) (Fig. 1C–IV). Pno1 expression in adult Sk seemed to be less abundant than in p10 Sk (comparative data not included). In lymphoid organs, the thymus continued to present high expression levels (Fig. 1C–V), while expression in the spleen remained moderate or low (Fig. 1C–III). Expression in the thymus occurred mainly in the cortex (Fig. 2A). Adult mouse BM cells displayed Pno1 signals both in x-ray film autoradiography (Fig. 2B–I) and emulsion autoradiography (Figs. 2B–II and III). BM in the Vb cavity showed labelled islands containing cells with small diameters (likely hematopoietic cells) in the midst of a meshwork of unlabelled connective tissue cells (Figs. 2B–II and III). The adrenal glands that are composed of amine- and peptide-producing medulla (Me), and steroid hormone-producing cortex (Cx) displayed Pno1 mRNA labelling in both regions, but predominantly in Cx (Figs. 2C–I, II and III).

### Generation of Pno1 Gene KO Mice

To understand Pno1 function, we generated Pno1 gene KO mice. The targeting strategy is depicted in Figure 3A. With the 3′ end probe, the WT allele after BamHI digestion gave an 18.6-kb band on Southern blotting, and the KO allele, a 7.7-kb band (Fig. 3B). Germline transmission was confirmed by Southern blotting of tail DNA, and WT and HET mice were thus identified (Fig. 3B, left panel). PCR was undertaken for routine genotyping of ear DNA. WT samples presented a 548-bp band, and HET samples, a 224-bp band (Fig. 3B, right panel).

To ascertain if Pno1 gene deletion affected its expression, we measured Pno1 mRNA levels in different tissues by RT-qPCR. As shown in Fig. 3C, HET samples from the Li, Th and Spl presented lower Pno1 mRNA levels compared to WT samples. Pno1 was previously selected for study because it was inducible after T cell activation (data not reported). We activated T cells with solid phase anti-CD3 and anti-CD28 monoclonal antibodies (mAbs), and quantified Pno1 mRNA levels at different time points (1, 2, 6, 12 and 24 h after the initiation of culture). As expected, WT T cells presented augmented Pno1 mRNA levels between 6 and 24 h after activation. On the other hand, HET T cells only up-regulated Pno1 mRNA to about half of that in WT T cells (Fig. 3D). The data in Figures 3C and 3D confirmed the gene deletion of Pno1 in HET mice. They also indicated that Pno1 expression was gene copy number-dependent, as HET cells only expressed half of Pno1 at the mRNA level compared to WT cells.

### Pno1 KO is Lethal in Embryos

We failed to generate any live Pno1 KO mice. Systemic tracking of fetus genotype in different gestation stages revealed that KO fetuses could only be found at e3.5 but not e6.5 (Table 1). Therefore, the embryos must have died between e3.5 and e6.5. We then cultured e1.5 embryos from HET male and HET female mating to observe their development in vitro. As depicted in Figure 4, up to e2.5, WT, HET and KO development seemed to be comparable. However, after e3.5, KO embryos stopped developing, while WT and HET embryos proceeded normally at this stage and beyond, indicating that Pno1 is vital in embryonic development.

### No Detectable Anomalies in the Immune System of Pno1 HET Mice

As Pno1 was prominently expressed in the thymus and was up-regulated in T cells upon their activation, we set out to investigate whether a lack of Pno1 causes immune system abnormalities. HET mice, which expressed Pno1 at about 50% the normal Pno1 level, were used for this purpose because no KO mice could be produced. There were no apparent lymphoid organ anomalies in HET mice in terms of size, weight and colour (data not reported). T, B, CD4 and CD8 sub-populations in the spleen and lymph nodes (LN) of HET mice were comparable to those in WT mice, as were thymocyte sub-populations, such as CD4CD8 double-negative, CD4CD8 double-positive, CD4 single-positive and CD8 single-positive cells (Fig. 5A and B).

Although HET T cells could only express Pno1 at about 50% the normal Pno1 level upon activation by anti-TCR mAb on solid phase, they proliferated as well as WT T cells (Fig. 5C). Activation markers, such as CD25 and CD69, were up-regulated in HET CD4 and CD8 T cells comparably to their WT counterparts (Fig. 5D). Therefore, it seems that 50% of normal Pno1 expression is sufficient to maintain T cell development and function.

We also assessed B cell subpopulations in the spleen and peritoneal cavity of WT and HET mice. In the spleen of WT and HET mice, there was no consistent difference in terms of the percentage of B220^+^CD21^hi^CD23^lo/−^ mantel zone B cells (Fig. 6A), or the percentage of B220^+^CD21^int/hi^CD23^hi^ follicular B cells (Fig. 6A). For B cells of the WT and HET mouse peritoneal exudates, there was no consistent difference in terms of the percentage of B220^+^CD23^−^CD5^+^IgM^hi^ B1a cells (Fig. 6B), B220^+^CD23^−^CD5^−^IgM^hi^ B1b (Fig. 6B) and B220^+^CD23^+^IgM^int/hi^ B2 cells (Fig. 6B). Spleen B cells from WT and HET mice proliferated comparably upon anti-IgM plus IL-4, LPS plus IL-4, anti-CD40 plus IL-4, or anti-CD40 plus LPS stimulation (Fig. 6C). The upregulation of B cell activation markers CD86 and CD80 in WT and HET spleen B cells 24 h after the above-described stimulation showed no apparent difference neither (Fig. 6D). We next examined the percentage of IgD^−^IgM^−^CD138^+^B220^lo/−^ isotype-switched plasmablast/plasma cells in the draining lymph nodes of WT and HET mice 21 days after immununization with chick type II collagen, but no significant difference was found (Fig. 6E). The serum collagen-specific Ab levels in these WT and HET mice 21 days after the chick type II collagen immunization showed no significant difference (Fig. 6F). Therefore, it seems that a 50% reduction of Pno1 level does not affect the B cells functions in vitro and in vivo.

### Normal Proteasome Activity in HET Tissues

Because Pno1 is involved in proteasome maturation, according to studies in yeasts, we questioned whether reduced Pno1 expression in HET mice leads to decreased proteasome activity in cells. We tested 3 major proteasome protease activities (i.e., chymotrypsin-like, trypsin-like and caspase-like proteases, but the caspase-like proteases activity is not detectable) in splenocytes and thymocytes of HET mice, and discerned that they were comparable to those of WT mice (Fig. 7A). We also measured the protein level of one subunit (β5) of the proteasome complex in the lungs, spleens, thymuses, livers and kidneys of HET mice, and found that it was comparable to that of WT tissues (Fig. 7B). Taken together, these data suggest that a 50% reduction of Pno1 expression does not elicit abnormal proteasome levels or activities.

### Generation of Pno1 Tg Mice

Since we could not obtain live Pno1 KO mice, and since HET mice manifested no apparent anomalies in general and in immune system and proteasome activities in particular, we wondered whether Pno1 over-expression in mice would reveal some phenotype. We generated Tg mice with β-actin promoter-driven Pno1 expression. The plasmid construct is shown in Figure 8A. Tail DNA was routinely genotyped by PCR, and Tg mice presented a 552-bp band (Fig. 8B). In Tg organs, such as the lung, kidney, thymus and spleen, Pno1 mRNA expression was universally heightened, although to different degrees (Fig. 8C). We also tested chymotrypsin-like and trypsin-like protease activities in the Tg thymus and spleen, and found that they were similar to those of their WT counterparts (Fig. 8D).

### Pno1 Tg Mice Manifest no Apparent Immune System Anomalies

Pno1 Tg mice were fertile and had no gross anomalies. Their immune organs, the thymus, LN and Spleen, were of normal size and cellularity (data not reported). T and B cell populations and CD4 and CD8 T cell populations in the spleen and LN were comparable to those of WT mice (Fig. 9A). In the Tg thymus, the different thymic sub-populations (e.g., CD4CD8 double-negative, CD4CD8 double-positive, CD4 single-positive and CD8 single-positive cells) were similar to those in the WT thmus (Fig. 9B). Pno1 over-expression did not compromise T cell proliferation caused by solid phase anti-CD3 mAb stimulation (Fig. 9C). Tg CD4 and CD8 T cells up-regulated their activation markers CD25 and CD69 in comparison to their WT counterparts upon activation (Fig. 9D). Therefore, excessive Pno1 expression does not seem to affect proteasome activity.

We also assessed B cell subpopulations in the spleen and peritoneal cavity of WT and Tg mice. In the spleen of WT and Tg mice, there was no consistent difference in terms of the percentage of B220^+^CD21^hi^CD23^lo/−^ mantel zone B cells (Fig. 10A), or the percentage of B220^+^CD21^int/hi^CD23^hi^ follicular B cells (Fig. 10A). For B cells of the WT and Tg mouse peritoneal exudates, there was no consistent difference in terms of the percentage of B220^+^CD23^-^CD5^+^IgM^hi^ B1a cells (Fig. 10B), B220^+^CD23^-^CD5^-^IgM^hi^ B1b (Fig. 10B) and B220^+^CD23^+^IgM^int/hi^ B2 cells (Fig. 10B). Spleen B cells from WT and Tg mice proliferated comparably upon anti-IgM plus IL-4, LPS plus IL-4, anti-CD40 plus IL-4, or anti-CD40 plus LPS stimulation (Fig. 10C). The upregulation of B cell activation markers CD86 and CD80 in WT and Tg spleen B cells 24 h after the above-described stimulation showed no apparent difference neither (Fig. 10D). We next examined the percentage of IgD^-^IgM^-^CD138^+^B220^lo/−^ isotype-switched plasmablast/plasma cells in the draining lymph nodes of WT and Tg mice 21 days after immununization with chick type II collagen, but not significant difference was found (Fig. 10E). The serum collagen-specific Ab levels in these WT and Tg mice 21 days after the chick type II collagen immunization showed no significant difference (Fig. 10F).

The results from this section suggest that a normal endogenous Pno1 expression level is sufficient for the tested T cell functions. Pno1 over-expression does not confer additional advantages.

### Pno1 Occurs in Complexes Larger than 26S Proteasomes

We wondered whether Pno1 is present in proteasome complexes in mammalian cells. For this purpose, we stably transfected L cells with a Pno1-expressing construct, pCEP-Pno1, in which Pno1 was fused with 3 copies of HA tags at the N-terminus. The transfected cells expressed greatly-enhanced Pno1 mRNA levels (Fig. 11A), but their proteasome activity remained similar to that of empty vector-transfected cells (Fig. 11B). L cell lysates were fractionated with glycerol density gradients, and the chymotrypsin-like proteasome activity of each fraction was quantified. As seen in Figure 11C, there were 2 peaks of enzymatic activity at fractions 13 and 17, corresponding to 20S and 26S proteasomes, respectively. Western blotting showed that the proteasome β5 subunit was present from fractions 13 to 21, with trace amounts found up to fraction 23 (Fig. 11D), confirming that proteasome complexes (20S and 26S) were in these fractions, which contained 2 peaks of proteasome activities (Fig. 11C). Interestingly, Pno1, as detected by anti-HA Ab, made a major appearance, starting from fraction 13 and ending at fraction 25, with trace amounts occurring until fraction 27 (Fig. 11E). Its location did not correspond exactly to that of 20S or 26S proteasomes based on both proteasome activity and β5 location, but obviously resided in fractions larger than 26S.

Is Pno1 then a part of ribosomes? We assessed small and large ribosome subunits in glycerol gradient fractions by anti-S6 and anti-L7 mAb Western blotting (Figs. 11F and 11G, respectively). S6 signals appeared from fraction 17 and ended at fraction 27, consistent with the size of the small 40S ribosome subunit. L7 signals presented from fraction 25 until fraction 27, consistent with the size of large 60S ribosome subunits. Fractions containing the small subunit overlapped with but were not identical to those of Pno1.

The results of this sedimentation study indicate that Pno1 does not co-localize exactly with proteasomes, small or large ribosome subunits, but seems to exist in complexes with sedimentation rates higher than 26S, probably between 30S to 40S, employing a low ribosome sedimentation rate as marker.

## Discussion

So far, all known functions of Pno1 are based on findings in yeasts, in which Pno1 is reportedly involved in both ribosome and proteasome biogenesis. In this study, we attempted to understand Pno1 function in mammalian cells, via a combination of ISH, gene KO, Tg and in vitro cell transfection techniques.

First of all, according to signal intensity in ISH, Pno1 mRNA belonged to a class of low-abundant species. In biological systems, such low-abundant species normally play regulatory roles so that a small change in their expression can be leveraged and amplified for much larger biological reactions at the molecular level.

The general expression pattern from the fetal stage to adulthood indicates that this gene is highly expressed in organs or tissues containing fast-proliferating cells, such as the e9 fetus, thymus at different ages, skin, bone marrow, intestine and testes. This is compatible with the presumed function of Pno1 in ribosome and proteasome neogenesis, because fast-growing cells need high rates of protein synthesis as well as protein degradation. However, Pno1 was also expressed at high levels in certain tissues with no proliferation or enhanced protein synthesis, such as neuroganglia (DRG, SG and TriG), OL and ONE in the brain, and Me of the Ad. On the other hand, although the adult liver is very active in protein synthesis, its Pno1 expression is very low. These observations raise an intriguing question that Pno1 might have important functions unrelated to either ribosome or proteasome neogenesis.

We observed that a 50% Pno1 reduction in HET cells or 4-5-fold Pno1 increase in Tg cells did not cause any disruption of embryonic development, proteasome activity, T cell development, B cell development, or in vitro T and B cell activation and proliferation. It is known that proteasome is important to plasma cell generation, and the proteasome inhibitors could induce apoptosis in malignant and primary plasma cells both in vitro and in vivo as a result of activation of the terminal unfolded protein response (UPR) [15], [16]. However, no anormaly was observed in HET and Tg mice in terms of plasma cell percentage and and their antibody production.

We introduced small interfering RNA into HET fibroblasts to further lower Pno1 expression to about 15% of the normal level, but the cells proliferated normally with proteasome activities comparable to those of their WT counterparts (data not included). However, a complete lack of Pno1 is disastrous: it led to arrest of embryonic development before e3.5, at which time embryo contain about 8–30 cells [17]. There are 2 possible interpretations of this observation: 1) around e2.5, Pno1 is critical for a certain developmental event; or 2) embryonic cells before eight cell stage depend on maternal Pno1 protein or mRNA for their vital functions until its concentration drops below the critical level because of cell division (i.e., less than 1/8 of the normal Pno1 cellular level on e2.5 due to cell division) and the cells then cease to function owing to a lack of Pno1. In support of this hypothesis, it is worth mentioning that maternal ribosomes are exhausted before the blastocyst stage at e3.5 [18], [19]. Regardless of what function Pno1 has, it is obviously a vital molecule with no redundancy and is absolutely required for cell function. Cells only need a small fraction of the normal Pno1 level to be functional. Excessive Pno1 does not bring any perceivable benefit, but without it, cells will die.

In yeasts, Pno1 has been implicated in ribosome and proteasome neogenesis. Whether it has similar functions in mammalian cells is not known. With density gradient sedimentation, we were able to identify fractions containing 20S and 26S proteasomes. However, the abundance of Pno1 in fractions containing proteasomes doesn’t match ecactly their protease activities, and Pno1 also appeared in fractions containing complexes with sedimentation rates higher than 26S. This at least indicates that Pno1 might not be a constitutive part of mature 20S or 26S proteasomes. In cells, the only macromolecules larger than proteasomes are ribosomes. In eukaryotic cells, mature ribosomes are 40S and 60S in size and derive from 90S precursors with some 66S and 43S intermediate products occurring during the maturation process [20], [21]. We used S6, a component of the small ribosome subunit, and L7, a component of the large ribosome subunit, as markers to establish fraction profiles of small 40S and large 60S ribosome subunits. The fraction profile of Pno1 did not fit either of them. Pno1-containing fractions appeared earlier than large ribosome-containing fractions. On the other hand, Pno1 overlapped with but appeared about 2 fractions later than small ribosome subunit-containing fractions. Several possibilities exist: 1) If Pno1 is involved in mammalian ribosome neogenesis, it is not a part of mature ribosome particles, but could transiently associate with certain pre-ribosome species and play a critical role in their assembly and maturation. Indeed, it is known that more than 200 molecules transiently associate with ribosomes during their neogenesis and are critical in their assembly. Pno1 could be one of them and an irreplaceable one at that. 2) As the size of Pno1-containing complexes is larger than 26S but smaller than 60S, the complexes might contain a 26S proteasome and some malformed ribosomes, smaller than 40S, to facilitate their elimination.

Because of early embryo lethality, we could not obtain sufficient KO cells to study the roles of Pno1 in proteasome or ribosome functions. As a consequence, we could not confirm whether a lack of Pno1 compromises mammalian proteasome or ribosome maturation or function. We immunoprecipitated Pno1 and tried to find proteasome subunits or ribosome subunits in precipitates, or *vice versa*, but met with little success (data not reported). This has raised an intriguing possibility that if Pno1 does link with proteasome and/or ribosome precursors, the association is transient and probably of low affinity. Additional studies with inducible gene KO in mice are needed to reveal the possible role of Pno1 in mammalian cells.
